# Peripheral Blood Biomarkers for Early Diagnosis, Severity, and Prognosis of Checkpoint Inhibitor-Related Pneumonitis in Patients With Lung Cancer

**DOI:** 10.3389/fonc.2021.698832

**Published:** 2021-07-13

**Authors:** Xinqing Lin, Haiyi Deng, Yilin Yang, Jianhui Wu, Guihuan Qiu, Suyang Li, Xiaohong Xie, Ming Liu, Zhanhong Xie, Yinyin Qin, Yong Song, Chengzhi Zhou

**Affiliations:** ^1^ State Key Laboratory of Respiratory Disease, National Clinical Research Centre for Respiratory Disease, Guangzhou Institute of Respiratory Health, First Affiliated Hospital, Guangzhou Medical University, Guangzhou, China; ^2^ Department of Respiratory and Critical Care Medicine, Jinling Hospital, Nanjing, China

**Keywords:** checkpoint inhibitor-related pneumonitis, immune checkpoint inhibitor, interleukin-6, lymphocyte, albumin, lung cancer

## Abstract

**Background:**

Checkpoint inhibitor-related pneumonitis (CIP) is a potentially fatal immune-related adverse event that occurs during treatment with immune checkpoint inhibitors (ICIs). However, the roles played by peripheral blood parameters in CIP development remain unclear. Here, we aimed to identify which blood biomarkers correlated with the development and prognosis of CIP in patients with lung cancer.

**Methods:**

We conducted a retrospective analysis of 87 patients with CIP (CIP group) and 87 patients without CIP (control group). Cytokines, blood routine, lactate dehydrogenase (LDH) and albumin (ALB) were collected at baseline (before ICIs), at onset of pneumonitis (in the CIP group), and before the last dose of ICI (in the control group). We compared the baseline values and changes over time in various blood parameters between the CIP and control groups. The CIP outcomes were collected and compared according to the median values of these parameters.

**Results:**

Squamous carcinoma (odds ratio [OR]: 3.02; *p* = 0.004) and ICI monotherapy (OR: 6.56; *p* = 0.004) correlated with a high risk of CIP. In the CIP group, interleukin (IL)-6 and platelet-to-lymphocyte ratio (PLR) at CIP were significantly increased relative to baseline. By contrast, IL-6 and PLR reduced over time in the control group. Significant decrease in absolute lymphocyte count (ALC) and increases in IL-10, neutrophil to lymphocyte ratio (NLR), and LDH levels were observed from baseline to CIP. No significant change in these parameters was observed in the control group relative to baseline. ALB decreased in both groups, but the decrease in the CIP group was greater (9.21% *vs*. 2.44%; *p* = 0.020). High IL-6 levels (OR: 5.23, 95% confidence interval [CI]: 1.15–23.86; *p* = 0.033), and low levels of ALB (OR: 0.16, 95% CI: 0.04–0.64; *p* = 0.009) measured at the time of CIP symptom onset were associated with severe pneumonitis. Low concentration of IL-6 (hazard ratio [HR]: 0.17, 95% CI: 0.03–0.95; *p* = 0.044) and high ALB levels (HR: 0.28, 95% CI: 0.08–0.94; *p* = 0.040) were correlated with favorable overall survival in CIP.

**Conclusions:**

Increase in IL-6, IL-10, NLR, PLR, and LDH levels or reduced ALC and ALB levels were associated with the occurrence of CIP in lung cancer patients. High IL-6 and low ALB levels at onset of CIP were related to severe grade and poor prognosis of CIP.

## Introduction

Immune checkpoint inhibitors (ICIs) provide enhanced survival benefits to patients with malignant tumors, including lung cancer (1, 2); however, ICIs sometimes cause a series of unique adverse events, known as immune-related adverse events (irAEs) (3). A review of 20 randomized controlled studies suggested that the incidence of fatal irAEs associated with programmed death-1 (PD-1)/programmed death-ligand 1 (PD-L1) inhibitors was 0.43%, among which checkpoint inhibitor-related pneumonitis (CIP) was the most common (4). A meta-analysis showed that lung cancer was more likely than other cancers to result in all-grade or high-grade CIP (5). CIP lacks typical clinical symptoms, and 1/3 of patients with CIP are asymptomatic at the time of onset (6). The delayed treatment of CIP patients may lead to disease aggravation. The overall survival (OS) of patients with CIP who do not recover or whose symptoms worsen is significantly shortened compared with those who recovered from CIP (7). Therefore, determining the risk factors associated with CIP and early CIP identification is crucial. Previous studies showed that age, smoking status, pre-existing lung diseases, and chest radiotherapy history might be related to CIP occurrence (8–10). However, the sample sizes of CIP patients in these studies are small, and whether other risk factors may exist is also worthy of further study.

Blood-based biomarkers have the advantages of minimally invasive, easy to collect, and reproducible. Studies have shown that C-reactive protein (CRP), interleukin (IL)-6, blood cell counts, and cytokine levels are associated with irAEs (11). Recent data suggest that the neutrophil to lymphocyte ratio (NLR) may be related to irAE onset, severity, and subsequent prognosis (12). Similarly, increased NLR values may contribute to the diagnosis of ICI-associated myocarditis (13). A recent study indicated that elevated IL-6, IL-10, and eosinophil levels might be indicators of skin-related irAEs (14). However, a few reports have examined the association between peripheral blood biomarkers and CIP occurrence. Previous reports have shown that an increased anti-CD74 autoantibody was correlated with CIP occurrence (15). However, these biomarkers are not included in routine clinical tests, and their determination requires special equipment.

Previous studies have suggested that the OS of patients with irAEs was significantly longer than that of patients without irAEs. However, in the subgroup analysis, CIP was not significantly associated with ICI efficacy (16, 17). Conversely, a study by Fukihara et al. suggested that OS was significantly shorter among patients with CIP than among those without CIP (18). Another study showed that grades 1–2 CIP was associated with favorable OS, whereas grades 3–4 CIP was not (19). The survival time for CIP patients varies greatly. Therefore, determining whether peripheral blood markers can be used to predict OS in patients with CIP remains necessary.

This study was designed to identify the potential risk factors in baseline clinical characteristics associated with the occurrence of CIP and to investigate the association between clinically accessible biomarkers in peripheral blood and the development or prognosis of CIP.

## Materials and Methods

### Patients

This retrospective, observational study was conducted at the First Affiliated Hospital of Guangzhou Medical University. Records for patients with unresectable stage III or IV primary lung cancer [according to the 2015 World Health Organization Classification of Lung Tumors (20)] treated with at least one dose of ICI between January 2016 and January 2021 were reviewed. Patients who developed CIP (CIP group) and randomly selected corresponding patients without CIP (control group) were included at a ratio of 1:1. Prior tuberculosis and bacterial and fungal infections in the lungs before immunotherapy were excluded. All procedures performed in this study involving human participants were in accordance with the Declaration of Helsinki (as revised in 2013). This study was approved by the local Ethics Committee of the First Affiliated Hospital of Guangzhou Medical University.

### Diagnosis of CIP

CIP was diagnosed by two experienced pulmonologists and one chest radiologist, based on the guidelines of the National Comprehensive Cancer Network, the American Society for Clinical Oncology, and the European Society for Medical Oncology (21–23). We defined CIP as new-onset infiltrates on thoracic imaging and/or clinical symptoms of cough, shortness of breath, or wheezing that is likely to be caused by ICIs, and excluded other etiologies. For patients considering the diagnosis of CIP, several examinations were performed in order to exclude other lung diseases (e.g. pulmonary infections and tumor progression), such as bronchoalveolar lavage culture, sputum cultures and laboratory tests (routine blood test, procalcitonin, tumor markers, arterial gas analysis, serous D-dimer and brain natriuretic peptide, etcetera). In addition, when patients with pulmonary infection had been a poor response to anti-infection treatment, CIP with pulmonary infection may be diagnosed. We compared pneumonitis extent and previous radiation field to exclude radiation-induced pneumonitis. If the diagnosis was not clear and the patient’s physical condition allowed, a lung biopsy would be performed.

### Data Collection and Outcome Assessment

The following information was retrospectively collected from each patient’s medical records: patient demographics, pre-existing lung disease, tumor histology, Eastern Cooperative Oncology Group Performance Status (ECOG PS), radiation therapy administrations, treatment data, and driver gene status. The ECOG PS was evaluated prior to ICI treatment. Driver gene status was tested before any anti-tumor treatments were applied. In the CIP group, we also collected the time course of CIP, maximum CIP grade, and CIP outcomes. The severity of CIP was graded according to the Common Toxicity Criteria for Adverse Events (CTCAE version 4.0). In the CIP group, OS was calculated from the date of CIP diagnosis until death or the last follow-up date (April 1, 2021).

Among patients with CIP, we collected peripheral blood parameters at two time points: baseline (prior to ICI treatment), and at the time of CIP diagnosis. In the control group, we recorded these parameters at two time points: baseline before starting ICI treatment and before the last dose of ICI. Peripheral blood parameters included IL-2, IL-4, IL-6, IL-10, interferon-gamma (IFN-γ), tumor necrosis factor-α (TNF-α), absolute neutrophil count (ANC), absolute lymphocyte count (ALC), absolute eosinophil count (AEC), platelet count (PLT), lactate dehydrogenase (LDH), and albumin (ALB). The NLR was calculated as ANC divided by ALC. The platelet-to-lymphocyte ratio (PLR) was calculated by dividing PLT by ALC.

### Statistical Analysis

Continuous variables were summarized as the median and interquartile range (IQR). Categorical data were summarized as the frequency (percentage). Differences in continuous variables at baseline were assessed using either an independent-samples t-test or the Mann–Whitney U test. Chi-square (χ^2^) or Fisher’s exact test was used to analyze categorical variables.

Logistic univariate analysis was used to determine which factors were associated with CIP. Multivariate logistic regression analysis was used to analyze those variables with *p*-value <0.1 in the univariate analysis to determine potential CIP risk factors. Changes in peripheral blood parameters over time were evaluated using a paired t-test or the Wilcoxon signed-rank test. The calculation of percentage change was performed as follows: (difference from baseline/baseline value) × 100. The Mann–Whitney U test was used to compare changes in blood parameters between the CIP and control groups. For those blood parameters with significant changes over time, the median value at the time of CIP diagnosis was used to perform logistic univariate and multivariate analyses to identify potential biomarkers associated with severe-grade CIP in the CIP group.

Finally, the Kaplan–Meier method was used to evaluate OS, with 95% confidence intervals (CIs), and the log-rank test was used to determine the significance of differences between two or more subgroups in CIP patients. A Cox proportional hazards model was used to identify prognostic factors associated with OS in the CIP group using multivariable survival analysis, including those variables with *p*-values <0.01 in the univariate analysis. Univariate and multivariate hazard ratios (HRs), with 95% CI values, were calculated.

Statistical analyses were conducted using IBM SPSS Statistics (Armonk, NY), version 25. All *p*-values were based on the two-sided hypothesis test, and a *p*-value <0.05 was considered significant.

## Results

### Participants

**Figure 1:**
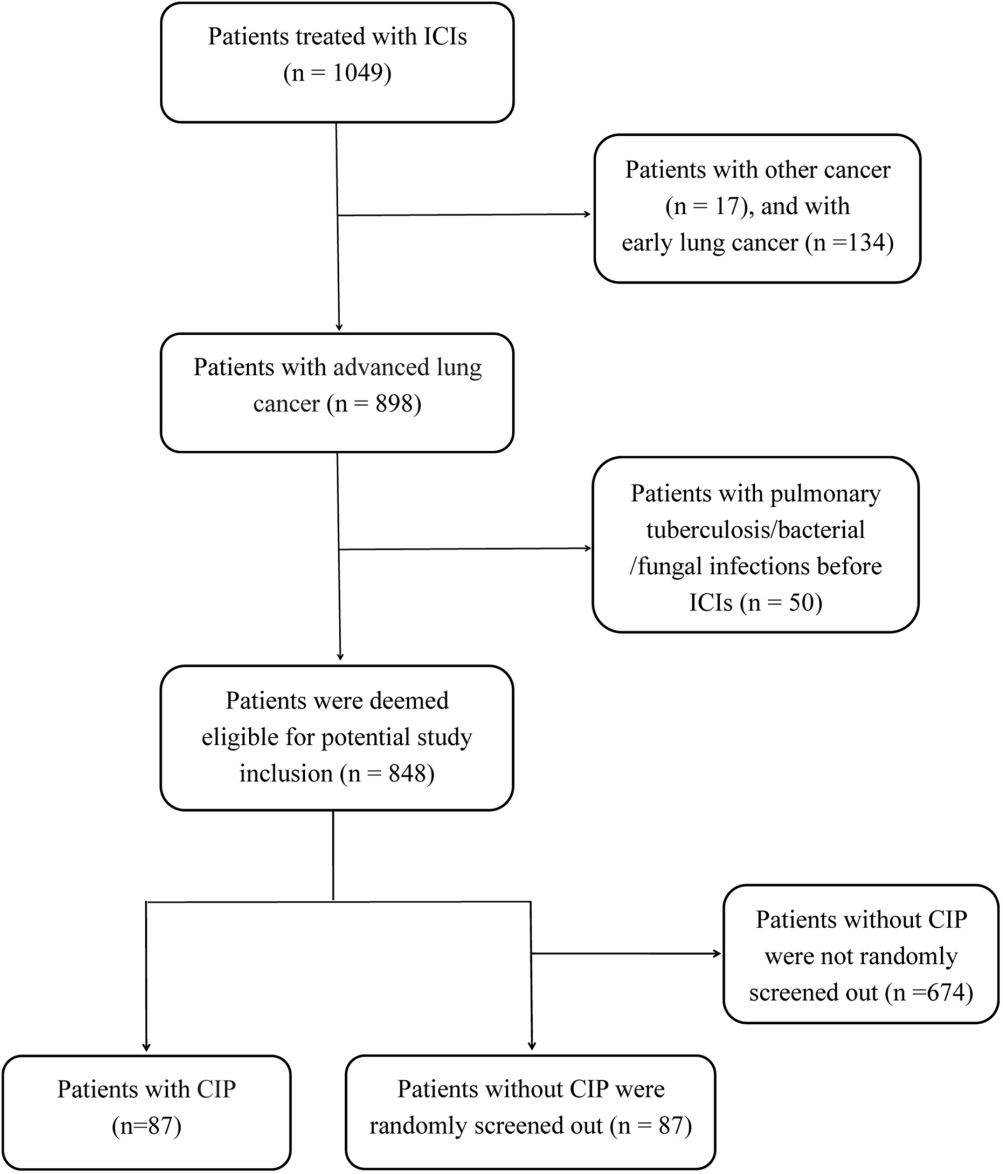
The flow chart of study design and patients inclusion. ICIs, immune checkpoint inhibitors; CIP, checkpoint inhibitor-related pneumonitis.

A total of 848 patients with advanced lung cancer who were treated with ICIs at our institution were deemed eligible for potential study inclusion. Finally, 87 patients (10.3%) who developed CIP (CIP group) and 87 randomly selected patients without CIP (control group) were included in the analysis (**Figure 1**). All patients were treated with PD-1 or PD-L1 inhibitors, with PD-1 inhibitors being more commonly used. The demographic characteristics were similar between the CIP and control groups (**Table 1**). However, the distributions of tumor types and treatment data among cases and controls were significantly different. Squamous cell carcinoma was the most common histologic type among the CIP group (42.5%), whereas adenocarcinoma (62.1%) was the most common type in the control group. In addition, combination therapy (including ICI + chemotherapy and ICI + chemotherapy + antiangiogenic drugs) was the predominant treatment type for both groups, but ICI monotherapy comprised a larger proportion (25.3%) of treatment types in the CIP group (*p <*0.001). Compared with the control group, the CIP group had a higher frequency of prior radiation (10.3% *vs*. 20.7%; *p* = 0.006).

**Table 1:** Baseline characteristics in advanced lung cancer patients treated with ICIs.

Variables	CIP group (n = 87)	Control group (n = 87)	P-value
Age			
Median (range)	65 (18–85)	62 (31–83)	0.20
<65, n (%)	42 (48.3)	52 (59.8)	0.17
≥65, n (%)	45 (51.7)	35 (40.2)	
Gender, n (%)			0.26
Male	73 (83.9)	66 (75.9)	
Female	14 (16.1)	21 (24.1)	
Smoking status, n (%)			0.76
Current/former	45 (51.7)	42 (48.3)	
Never	42 (48.3)	45 (51.7)	
Preexisting lung diseases, n (%)	15 (17.2)	14 (16.1))	1
Histologic type, n (%)			**<0.001**
Squamous	37 (42.5)	13 (14.9)	
Adenocarcinoma	22 (25.3)	54 (62.1)	
NOS	3 (3.4)	3 (3.4)	
SCLC	10 (11.5)	9 (10.3)	
Others	15 (17.2)	8 (9.2)	
ECOG PS			0.08
0–1	82 (94.3)	74 (85.1)	
≥2	5 (5.7)	13 (14.9)	
Prior radiation, n (%)	18 (20.7)	9 (10.3)	0.06
EGFR/ALK mutation (initial biopsy/pre-TKI)	6 (6.9)	3 (3.4)	0.28
Treatment line, n (%)			
1	62 (71.3)	70 (80.5)	0.11
≥2	25 (28.7)	17 (19.5)	
ICI type, n (%)			1
PD-1	82 (94.3)	82 (94.3)	
PD-L1	5 (5.7)	5 (5.7)	
Treatment data, n (%)			**<0.001**
Monotherapy	22 (25.3)	3 (3.4)	
Combination therapy	65 (74.7)	84 (96.6)	

Bold values indicate p < 0.05; NOS, not otherwise specified; SCLC, small cell lung cancer; ECOG PS, Eastern Cooperative Oncology Group performance status; EGFR, epidermal growth factor receptor gene; ALK, anaplastic lymphoma kinase; TKI, tyrosine kinase inhibitors; ICI, immune checkpoint inhibitors; PD-1, programmed cell death protein-1; PD-L1, programmed death ligand-1; CIP, checkpoint inhibitor-related pneumonitis.

Among the 87 patients with CIP, the median age was 65 years (range: 18–85 years), and 83.9% were men. The median time from the initial administration of ICIs to the development of CIP was 3.8 months (range: 0.2–20.7 months). Among the CIP patients, 38 patients (43.7%) had severe (grades 3–5) CIP. The baseline TNF-α level of patients with CIP tended to be lower than that among those without CIP, but no significant difference was observed (*p* = 0.06; **Supplementary Table 1**).

In the univariate and multivariate analysis (**Table 2**), squamous carcinoma (odds ratio [OR]: 3.02, 95% CI: 1.41–6.43; *p* = 0.004) and ICI monotherapy (OR: 6.56, 95% CI: 1.79–23.98; *p* = 0.004) correlated independently and significantly with the occurrence of CIP.

**Table 2:** Univariate and multivariate logistic regression analysis for the risk factors of CIP.

Variables	Univariate Analysis		Multivariate Analysis	
	OR (95% CI)	P-value	OR (95% CI)	P-value
Age (≥65 *vs*. <65)	1.59 (0.87–2.9)	0.13	–	–
Gender (female *vs*. male)	1.67 (0.78–3.5)	0.19	–	–
Smoking (current or former *vs*. never)	1.15 (0.63–2.1)	0.65	–	–
ECOG PS (≥2 *vs*. <2)	0.35 (0.12–1.02)	0.054	0.38 (0.12–1.17)	0.09
Prior radiation	2.26 (0.95–5.36)	0.06	1.95 (0.75–5.02)	0.19
Histology (squamous *vs*. non-squamous)	3.86 (1.89–7.87)	**<0.001**	3.02 (1.41–6.43)	**0.004**
Treatment line (≥2nd *vs*. 1st)	1.66 (0.82–3.36)	0.16	–	–
Treatment (monotherapy *vs*. combination)	9.48 (2.72–33.04)	**<0.001**	6.56 (1.79–23.98)	**0.004**

Bold values indicate p < 0.05; CIP, checkpoint inhibitor-related pneumonitis; ECOG PS, Eastern Cooperative Oncology Group performance status; OR, odds ratio; CI, confidence interval.

### Correlation of Biomarkers With CIP

**Figure 2:**
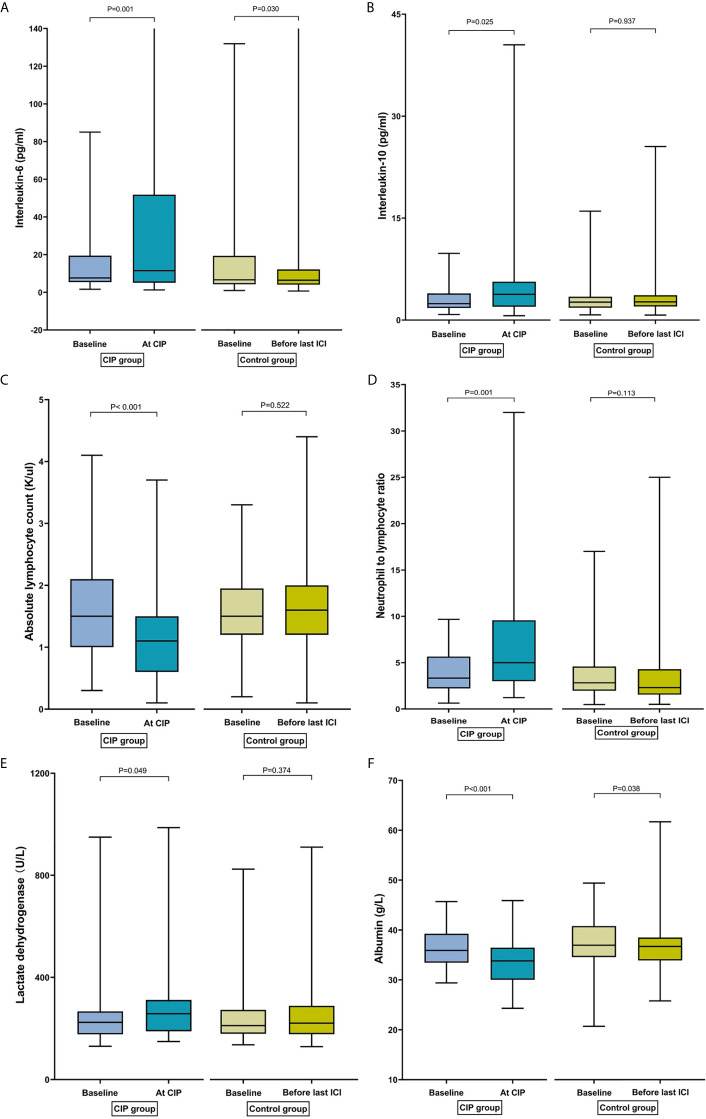
Bar plots of peripheral blood parameters in patients with checkpoint inhibitor-related pneumonitis (CIP) and controls at different time points. **(A)** Interleukin-6. **(B)** Interleukin-10. **(C)** Absolute lymphocyte count. **(D)** Neutrophil-to-lymphocyte ratio. **(E)** Lactate dehydrogenase. **(F)** Albumin.

IL-6 increased significantly from baseline to CIP [7.62 pg/ml (IQR: 5.42–17.46) to 11.81 pg/ml (IQR: 5.10–63.34); p = 0.001] in the CIP group. By contrast, a significant decrease in IL-6 levels was observed over time [6.66 pg/ml (IQR: 4.24–19.38) to 6.45 pg/ml (IQR: 3.92–12.79); p = 0.030] in the control group (**Figure 2A**). Similarly, the median levels of IL-10 at baseline and CIP were 2.41 and 3.79 pg/ml (p = 0.025), respectively in the CIP group, and no change in the IL-10 over time was observed among controls (p = 0.94; **Figure 2B**).

In the CIP group, ALC decreased significantly from baseline to CIP presentation [1.50 K/µl (IQR: 1.00–2.08) to 1.15 K/µl (IQR: 0.63–1.50); *p <*0.001]. However, ALC did not change over time in the control group [1.50 K/µl (IQR: 1.20–2.10) to 1.60 K/µl (IQR: 1.20–2.00); *p* = 0.52] (**Figure 2C**). Among CIP cases, a significant increase in NLR was observed from baseline to CIP presentation [3.58 (IQR: 2.44–6.79) to 5.38 (IQR: 3.07–10.32); *p* = 0.001]. However, no change in NLR over time was observed in the control group [2.82 (IQR: 1.97–4.58) to 2.31 (IQR: 1.55–4.29); *p* = 0.11] (**Figure 2D**). Similarly, an increase in the PLR was observed from baseline to CIP development [179.70 (IQR: 123.09–331.75) to 263.76 (IQR: 152.65–432.77); *p* = 0.008]. By contrast, the PLR decreased significantly from baseline to before the last ICI dose [161.11 (IQR: 121.05–231.58); *p* = 0.042] in the control group.

LDH of patients with CIP increased significantly from baseline to CIP [223.80 U/L (IQR, 177.03–398.93) to 257.85 U/L (IQR, 189.03–311.83); *p* = 0.049]. Nevertheless, there was no change in the LDH over time among patients without CIP (*p* = 0.37; **Figure 2E**). There was a significant decrease in the ALB from baseline to CIP [35.85 g/L (IQR, 33.45–39.25) to 33.80 g/L (IQR, 30.00–36.45); *p <*0.001]. Median ALB concentration was also comparable over time (36.95 *vs*. 36.70 g/L; p = 0.038) at baseline among cases. However, the decrease of ALB was higher in the CIP group than in the control group (9.21% *vs*. 2.44%; *p* = 0.020) (**Figure 2F**).

In the CIP group, IL-2, IL-4, IFN-γ, TNF-α, ANC, AEC, and PLT had no significant changes from baseline to presentation with CIP (**Supplementary Table 2**). No matter in the experimental group or the control group, in the subgroup analysis, changes in IL-6, IL-10, ALC, NLR, PLR, LDH and ALB over time were not statistically significant between the squamous carcinoma and non-squamous carcinoma groups, or between the combination therapy and monotherapy groups.

### Correlation of Biomarkers and Severe CIP

During follow-up, severe CIP occurred in 38 cases (43.7%). In the logistic univariate analysis, high IL-6, NLR, and PLR levels were associated with severe pneumonitis (grade 3 or higher) in the CIP group. By contrast, high concentrations of ALC and ALB were negatively correlated with severe pneumonitis. Multivariate regression analysis showed that high levels of IL-6 (OR: 5.23, 95% CI: 1.15–23.86; *p* = 0.033) and low levels of ALB (OR: 0.16, 95% CI: 0.04–0.64; *p* = 0.009) were significantly associated with CIP (**Table 3**).

**Table 3:** Univariate and multivariate logistic regression analysis for the risk factors of grades 3–4 CIP in the CIP group.

Variables	Univariate Analysis		Multivariate Analysis	
	OR (95% CI)	P-value	OR (95% CI)	P-value
IL-6 (≥11.81 *vs*. <11.81)	5.18 (1.73–15.49)	**0.003**	5.23 (1.15–23.86)	**0.033**
IL-10 (≥3.79 *vs*. <3.79)	2.62 (0.97–7.04)	0.057	1.85 (0.45–7.63)	0.39
ALC (≥1.15 *vs*. <1.15)	0.19 (0.07–0.52)	**0.001**	0.19 (0.03–1.08)	0.06
NLR (≥5.38 *vs*. <5.38)	7.23 (2.58–20.24)	**<0.001**	1.28 (0.25–6.70)	0.77
PLR (≥263.76 *vs*. <263.76)	2.94 (1.19–7.29)	**0.020**	1.76 (0.36–8.60)	0.48
LDH (≥257.85 *vs*. <257.85)	1.83 (0.73–4.58)	0.19	–	–
ALB (≥33.80 *vs*. <33.80)	0.18 (0.07–0.47)	**<0.001**	0.16 (0.04–0.64)	**0.009**

Bold values indicate p < 0.05; CIP, checkpoint inhibitor-related pneumonitis; IL-6, interleukin-6; IL-10, interleukin-10; the units for IL-6 and IL-10 are both pg/ml; ALC, absolute lymphocyte count, the unit for ALC is K/μl; NLR, neutrophil to lymphocyte ratio; PLR, platelet-to-lymphocyte ratio; LDH, lactate dehydrogenase, the unit for LDH is U/L; ALB, albumin, the unit for ALB is g/L; OR, odds ratio; CI, confidence interval.

### Correlation of Biomarkers and Overall Survival

**Figure 3:**
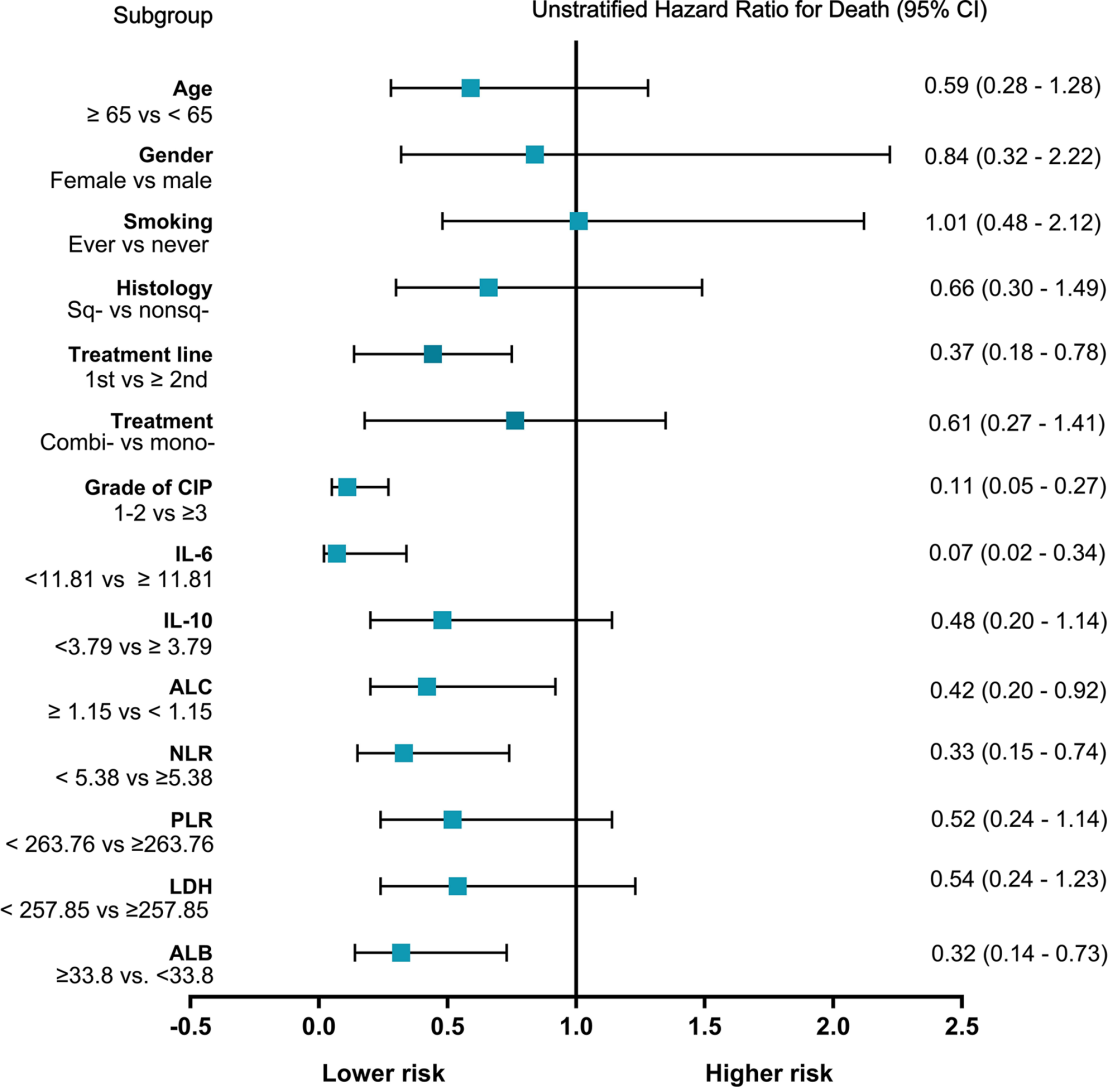
Forest plot of subgroup analyses of prognostic factors for overall survival of checkpoint inhibitor pneumonitis (CIP). Sq-, squamous; nonsq-, nonsquamous; combi-, combination; mono-, monotherapy; IL-6, interleukin-6; IL-10, interleukin-10; the units for IL-6 and IL-10 are both pg/ml; ALC, absolute lymphocyte count, the unit for ALC is K/μl; NLR, neutrophil to lymphocyte ratio; PLR, platelet-to-lymphocyte ratio; LDH, lactate dehydrogenase, the unit for LDH is U/L; ALB, albumin, the unit for ALB is g/L; CI, confidence interval.

**Figure 4:**
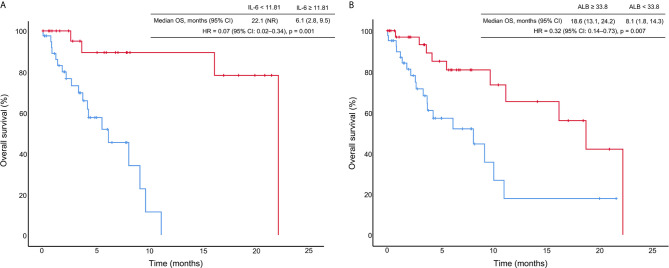
Kaplan–Meier curves of overall survival (OS) stratified by interleukin-6 (IL-6) levels **(A)** and albumin (ALB) concentration **(B)**. HR, hazard ratio; CI, confidence interval; the unit for IL-6 is pg/ml; the unit for ALB is g/L.

Among all patients with CIP, the median OS was 11.1 months (95% CI: 4.4–17.8 months), and the one-year survival rate was 46.5%. We generated a univariate Cox proportional hazards regression model of variables measured at the time of pneumonitis diagnosis. The results showed that CIP grade, and IL-6, ALC, NLR, and ALB levels were significantly correlated with OS (**Table 4** and **Figure 3**). The median OS was significantly different according to treatment line (1st *vs* ≥2nd: 18.6 *vs* 5.5 months; HR: 0.37, 95% CI: 0.18–0.78; *p* = 0.009), CIP grade (1–2 *vs*. ≥3: 22.1 *vs* 3.7 months; HR: 0.11, 95.0% CI: 0.05–0.27; *p <*0.001), IL-6 (<11.81 *vs*. ≥11.81: 22.1 *vs* 6.1 months; HR: 0.07, 95.0% CI: 0.02–0.34; *p* = 0.001) (**Figure 4A**), ALC (≥1.15 *vs*. <1.15: 10.9 *vs*. 5.5 months; HR: 0.42, 95% CI: 0.20–0.92; *p* = 0.029), NLR (<5.38 *vs*. ≥5.38: 22.1 *vs*. 9.1 months; HR: 0.33, 95.0% CI: 0.15–0.74; *p* = 0.007), and ALB (≥33.80 *vs*. <33.80: 18.6 *vs*. 8.1 months; HR: 0.32, 95% CI: 0.14–0.73; *p* = 0.007) (**Figure 4B**).

**Table 4:** Cox proportional hazards regression analysis of clinical factors associated with overall survival of CIP patients.

Variables	Univariate Analysis		Multivariate Analysis	
	HR (95% CI)	P-value	HR (95% CI)	P-value
Age (≥65 *vs*. <65)	0.59 (0.28–1.28)	0.18	–	–
Gender (female *vs*. male)	0.84 (0.32–2.22)	0.72	–	–
Smoking (current or former *vs*. never)	1.01 (0.48–2.12)	0.99	–	–
Histology (squamous *vs*. non-squamous)	0.66 (0.30–1.49)	0.30	–	–
Treatment line (1st *vs*. ≥2nd)	0.37 (0.18–0.78)	**0.009**	0.61 (0.21–1.76)	0.36
Treatment (combination *vs*. monotherapy)	0.61 (0.27–1.41)	0.25	–	–
Grade of CIP (1–2 *vs*. ≥3)	0.11 (0.05–0.27)	**<0.001**	0.41 (0.11–1.56)	0.19
IL-6 (<11.81 *vs*. ≥11.81)	0.07 (0.02–0.34)	**0.001**	0.17 (0.03–0.95)	**0.044**
IL-10 (<3.79 *vs*. ≥3.79)	0.48 (0.20–1.14)	0.057	0.75 (0.27–2.04)	0.57
ALC (≥1.15 *vs*. <1.15)	0.42 (0.20–0.92)	**0.029**	0.29 (0.05–1.50)	0.14
NLR (<5.38 *vs*. ≥5.38)	0.33 (0.15–0.74)	**0.007**	1.11 (0.25–4.90)	0.89
PLR (<263.76 *vs*. ≥263.76)	0.52 (0.24–1.14)	0.09	1.35 (0.40–4.58)	0.63
LDH (<257.85 *vs*. ≥257.85)	0.54 (0.24–1.23)	0.14	–	–
ALB (≥33.80 *vs*. <33.80)	0.32 (0.14–0.73)	**0.007**	0.28 (0.08–0.94)	**0.040**

Bold values indicate p < 0.05; CIP, checkpoint inhibitor-related pneumonitis; IL-6, interleukin-6; IL-10, interleukin-10; the units for IL-6 and IL-10 are both pg/ml; ALC, absolute lymphocyte count, the unit for ALC is K/μl; NLR, neutrophil to lymphocyte ratio; PLR, platelet-to-lymphocyte ratio; LDH, lactate dehydrogenase, the unit for LDH is U/L; ALB, albumin, the unit for ALB is g/L; HR, hazard ratio; CI, confidence interval.

In the multivariate Cox proportional hazards regression model, only IL-6 (<11.81 *vs*. ≥11.81: HR = 0.17, 95% CI: 0.03–0.95; *p* = 0.044) and ALB (≥33.80 *vs*. <33.80: HR = 0.28, 95% CI: 0.08–0.94; *p* = 0.040) were significantly and independently correlated with OS in patients with CIP (**Table 4**).

## Discussion

This real-world, retrospective, observational study suggested that the histologic cancer type and ICI monotherapy may be risk factors of CIP occurrence. We found that IL-6, IL-10, ALC, NLR, PLR, LDH, and ALB levels changed significantly over time in patients with CIP. In addition, IL-6 and ALB levels at the time of CIP diagnosis were significantly correlated with severity and OS in CIP patients.

In our study, the overall CIP incidence was estimated at 10.3%, and 4.5% of patients developed grade 3 or higher CIP, which were larger proportions than those reported in previous clinical trials (24) but were consistent with a previous real-world study (10). The incidence of prior radiation was higher in CIP group than those in control group (20.7% *vs*. 10.3%; p = 0.06). In univariate logistic regression analysis, prior radiation tended to be associated with CIP (OR: 2.26, 95% CI: 0.95–5.36; p = 0.06). Multiple studies have shown that the history of prior radiotherapy could increase the risk of developing pneumonitis (8, 25). Our logistic regression analyses suggested that squamous carcinoma was associated with a high incidence of CIP. A previous study also reported that squamous carcinoma might be a risk factor for pneumonitis (26). One study showed that obstructive pneumonia may increase the risk of CIP (27). Most squamous cell carcinomas are central lung cancer, and obstructive pneumonia occurs more frequently, which may explain the increased incidence of pneumonitis in patients with squamous carcinoma. The association between pathological cancer types and CIP occurrence is worthy of further study. Our finding of a higher (OR: 6.56, 95% CI: 1.79–23.98; p = 0.004) CIP incidence among patients treated with ICI monotherapy was consistent with the findings of a recent meta-analysis (28), which showed that ICI monotherapy was associated with a higher risk of CIP (OR: 2.14, 95% CI: 1.12–4.80), compared with ICIs plus chemotherapy. This may be partly explained by cytotoxic chemotherapy drugs that can cause immunosuppression, and possibly the use of glucocorticoids as a pretreatment of chemotherapy, which may suppress the immune system as well as treat certain underlying lung diseases (e.g. asthma and chronic obstructive pulmonary disease) (28). In addition, antiangiogenic drugs (e.g. bevacizumab) could reduce vascular permeability and pulmonary exudation, which may contribute to the recovery of early pneumonitis (29). A case report showed that the addition of nintedanib to immunotherapy may prevent CIP (30).

With the development of irAEs, increased serum IL-6 and IL-10 levels have been demonstrated in case reports and retrospective studies with small samples (31–36). However, changes in the levels of these cytokines have only been reported in individual CIP cases. A case study showed a significant increase in IL-6 at the onset of CIP (37). Our study represents the first retrospective study to explore the relationship between cytokines and CIP development. We found that IL-6 and IL-10 levels increased significantly at CIP onset compared with those at baseline. However, the IL-10 levels remained unchanged, and the IL-6 levels decreased in patients without CIP over time. Elevated IL-6 was an independent biomarker for CIP severity and was an independent predictor for early death. In addition, high levels of IL-10 tended to be associated with severe CIP (p = 0.057). A study showed that the lymphocytes in the alveolar lavage fluid (BAL) of patients with CIP increased, predominantly CD4+ T helper (Th) lymphocytes (38). Th2 cells, an important subset of CD4 + cells, can produce cytokines (such as IL-4, IL-5, IL-6, IL-9, IL-10, and IL-13), which in turn leads to excessive inflammation (39). These data supported the hypothesis that the excessive activation and proliferation of T cells cause an excessive cascade of cytokine release, which, in turn, causes an excessive immune response, leading to the occurrence of CIP. A previous case report showed that a patient developed severe cytokine release syndrome (CRS) after treatment with a PD-1 inhibitor (40). Thus, severe CIP may be related to CRS, which is a systemic inflammatory response caused by the release of inflammatory cytokines after the activation of monocytes, macrophages, and other lymphocyte populations, and elevated IL-6 plays a key role in this process (41). Stroud et al. reported that 27/34 patients with irAEs had improved clinical symptoms after receiving tocilizumab (IL-6 inhibitors) (42). Thus, IL-6 inhibitors may be an option for individualized treatment of CIP patients.

We observed that peripheral blood ALC values decreased from baseline to CIP, whereas no change was observed in the control group. A previous study suggested that a higher baseline ALC level (>2000 cells/mL) was a risk factor for irAE (43). In univariate analysis, low ALC levels were correlated with severe pneumonitis. Fujisawa et al. reported that a decrease in ALC values was associated with the incidence of grades 3–4 CIP in melanoma patients treated with nivolumab (44). This phenomenon may be caused by the large number of lymphocytes transported from the blood that infiltrate the focus of pneumonitis, resulting in a reduction of ALCs in the circulating pool, especially in severe patients, which is manifested as reduced peripheral blood ALC values (45). CIP should be distinguished from pulmonary infections, especially bacterial pneumonia. Bacterial pneumonia is primarily characterized by increased neutrophils; however, in our study, CIP patients did not present with increased neutrophils, and changes in the NLR appeared to primarily be due to a decrease in lymphocytes. Therefore, decreased ALC values may represent an indicator that can be used to differentiate CIP from bacterial pneumonia.

In our study, NLR and PLR increased significantly in CIP compared with baseline values. In univariate analysis, the observed increases in these two biomarkers at the time of CIP symptom onset were associated with CIP severity. No previous data have examined the role of PLR in CIP detection. A recent report (12) by Matsukane et al. analyzed NLR fluctuations in solid tumors and found that increased NLR was significantly associated with the occurrence of irAEs, especially in pneumonitis. They also indicated that elevated NLR levels at the time of CIP diagnosis were correlated with the occurrence of high-grade CIP. Conversely, a study showed that NLR and PLR were not associated with irAEs but were associated with the response to ICI treatment (31). However, this study only included patients treated with cytotoxic T-lymphocyte-associated protein 4 (CTL-4) inhibitors and did not analyze specific organs.

Multiple studies have shown that NLR and PLR are associated with OS in lung cancer patients treated with ICIs (46–48). However, the relationship between these indicators and the OS of patients with CIP is rarely reported. The univariate analysis showed that elevated NLR and low ALC levels at the time of initial CIP symptom onset were associated with shorter OS in patients with CIP. In a previously published study, compared with patients with a rapid decrease in elevated NLR, those patients who maintain elevated NLR had a poorer OS (12).

Studies have reported that damaged lung tissue cells release LDH, leading to increased serum LDH levels and suggesting that elevated LDH may serve as an indicator of acute lung injury (49–52). However, whether LDH is elevated in CIP has not yet been reported. Our study found that LDH was significantly higher in CIP than at baseline. A previous study suggested that patients with LDH levels greater than twice the upper limit of the normal tended to have a reduced risk of severe irAEs than patients with normal LDH levels (53). However, our study found no correlation between baseline LDH and the occurrence of CIP. Additionally, no correlation was observed between LDH and the severity of CIP.

In the current study, decreased ALB levels were associated with CIP development. A previous study showed that low ALB level was a risk factor for CIP. CIP may result in the release of both proinflammatory and inflammatory cytokines, which increase capillary permeability and promote the entry of cell and plasma solutes (such as ALB) into lesion tissue, increasing the interstitial volume and changing the distribution of ALB, which manifest as a decrease in serum ALB (54). In the multivariate analysis, high ALB levels were negatively correlated with severe pneumonitis (OR: 0.16, 95% CI: 0.04–0.64). In addition, low ALB level was a predictor of poor OS. Consistent with a previous study, these results suggested that low serum ALB may serve as a biomarker of inflammation severity and was associated with reduced quality of life and longevity (54).

These data indicate that the measurement of these indicators could be performed when CIP is clinically suspected, particularly when other measurement methods, such as chest CT or chest X-ray, are not available or are inconclusive. In addition, these indicators may help identify patients who are at risk of severe CIP and may be used to predict CIP prognosis. However, this study has some limitations. First, this study is a real-world retrospective study. Second, we did not monitor all changes in these blood parameters from the beginning of ICI to the onset of CIP. Third, CIP was diagnosed by symptoms and radiology, and only 19.5% of patients were confirmed by histopathology.

In conclusion, squamous carcinoma and ICI monotherapy may represent risk factors for CIP development. Increases in IL-6, IL-10, NLR, PLR, and LDH levels or reductions in ALC and ALB levels during ICI treatment may also serve as biomarkers for early diagnosis of CIP. High levels of IL-6 and low concentrations of ALB at the time of initial onset of CIP symptoms were predictive of severe pneumonitis. Importantly, high IL-6 or low ALB levels could be applied to improve risk stratification in pneumonitis.

## Data Availability Statement

The raw data supporting the conclusions of this article will be made available by the authors, without undue reservation.

## Ethics Statement

The studies involving human participants were reviewed and approved by Institutional Review Board of the First Affiliated Hospital of Guangzhou Medical University (Guangzhou, Guangdong, China). The patients/participants provided their written informed consent to participate in this study.

## Author Contributions

XL, HD, and CZ designed the study. HD, YY, JW, GQ, and SL collected the patients’ data. XL, HD, XX, ML, ZX, and YQ analyzed the data. XL, HD, YY, JW, and CZ drafted and revised the manuscript. All authors contributed to the article and approved the submitted version.

## Funding

This study was supported by Fundamental and Applied Fundamental Research Project of City-School (Institute) Joint Funding Project, Guangzhou Science and Technology Bureau (202102010345, 202102010357), and Zhongnanshan Medical Foundation of Guangdong Province (ZNSA-2020003).

## Conflict of Interest

The authors declare that the research was conducted in the absence of any commercial or financial relationships that could be construed as a potential conflict of interest.
