# Supplementary material for: Peripheral Blood Biomarkers for Early Diagnosis, Severity, and Prognosis of Checkpoint Inhibitor-Related Pneumonitis in Patients With Lung Cancer
Source: Front Oncol. 2021 Jul 13;11:698832. doi: 10.3389/fonc.2021.698832 (PMC8313853; doi:10.3389/fonc.2021.698832)
Supplement: Supplementary file 2 [file Table_1.docx]

Supplementary Table 1. Baseline peripheral blood biomarkers for the CIP and control groups

| Variables | Control group | | | CIP group | | | P-value |
| --- | --- | --- | --- | --- | --- | --- | --- |
|  | Median | IQR 1 | IQR 3 | Median | IQR 1 | IQR 3 |  |
| IL-2, pg/mL | 0.87 | 0.59 | 1.70 | 0.74 | 0.53 | 1.36 | 0.17 |
| IL-4, pg/mL | 1.76 | 0.95 | 2.97 | 1.62 | 1.01 | 2.44 | 0.81 |
| IL-6, pg/mL | 6.66 | 4.24 | 19.38 | 7.62 | 5.42 | 17.46 | 0.91 |
| IL-10, pg/mL | 2.64 | 1.82 | 3.47 | 2.41 | 1.77 | 3.91 | 0.87 |
| IFN-γ, pg/mL | 1.18 | 0.77 | 2.15 | 0.98 | 0.38 | 1.81 | 0.06 |
| TNF-α, pg/mL | 1.21 | 0.72 | 2.48 | 1.03 | 0.61 | 2.07 | 0.21 |
| ANC, K/μL | 4.60 | 3.33 | 6.00 | 5.05 | 3.60 | 6.92 | 0.29 |
| ALC, K/μL | 1.50 | 1.20 | 2.10 | 1.50 | 1.00 | 2.08 | 0.35 |
| AEC, K/μL | 0.20 | 0.10 | 0.30 | 0.14 | 0.10 | 0.29 | 0.32 |
| PLT, K/μL | 258.00 | 205.50 | 311.00 | 269.50 | 198.00 | 383.25 | 0.68 |
| NLR | 2.82 | 1.97 | 4.58 | 3.58 | 2.44 | 6.79 | 0.12 |
| PLR | 161.11 | 121.05 | 231.58 | 179.70 | 123.09 | 331.75 | 0.28 |
| LDH, U/L | 211.70 | 180.00 | 275.55 | 223.80 | 177.03 | 398.93 | 0.49 |
| ALB, g/L | 36.95 | 34.55 | 40.80 | 35.85 | 33.45 | 39.25 | 0.12 |

IL, interleukin; IFN-γ, interferon-gamma; TNF-α, tumor necrosis factor-α; ANC, absolute neutrophil count; ALC, absolute lymphocyte count; AEC, absolute eosinophil count; PLT, platelet count; NLR, neutrophil to lymphocyte ratio; PLR, platelet-to-lymphocyte ratio; LDH, lactate dehydrogenase; ALB, albumin.

Supplementary Table 2. Peripheral blood biomarkers at baseline and at the onset of CIP or before the last ICI dose for the CIP and control groups.

| Variables | Control group | | | CIP group | | |
| --- | --- | --- | --- | --- | --- | --- |
|  | Baseline (median) | At CIP (median) | P-value | Baseline (median) | Before last ICI (median) | P-value |
| IL-2, pg/mL | 0.87 | 0.96 | 0.89 | 0.74 | 0.75 | 0.79 |
| IL-4, pg/mL | 1.76 | 1.09 | 0.01 | 1.62 | 0.96 | 0.057 |
| IFN-γ, pg/mL | 1.18 | 1.79 | 0.02 | 0.98 | 1.25 | 0.51 |
| TNF-α, pg/mL | 1.21 | 1.60 | 0.16 | 1.03 | 1.03 | 0.89 |
| ANC, K/μL | 4.60 | 3.60 | 0.004 | 5.05 | 5.50 | 0.37 |
| AEC, K/μL | 0.20 | 0.10 | 0.94 | 0.14 | 0.10 | 0.21 |
| PLT, K/μL | 258.00 | 217.00 | 0.001 | 269.50 | 268.00 | 0.22 |

IL, interleukin; IFN-γ, interferon-gamma; TNF-α, tumor necrosis factor-α; ANC, absolute neutrophil count; AEC, absolute eosinophil count; PLT, platelet count.
